# Association of Chronic Periodontitis with Hemorrhagic Stroke: A Systematic Review and Meta-Analysis

**DOI:** 10.1055/s-0044-1793844

**Published:** 2024-12-10

**Authors:** Athanasios Tsimpiris, Ioannis Tsolianos, Andreas Grigoriadis, Zoi Tsimtsiou, Dimitrios G. Goulis, Nikolaos Grigoriadis

**Affiliations:** 1 Dental Sector, 424 General Military Training Hospital, Thessaloniki, Greece; 2 Dental School, Faculty of Health Sciences, Aristotle University of Thessaloniki, Thessaloniki, Greece; 3 Department of Preventive Dentistry, Periodontology and Implant Biology, Dental School, Faculty of Health Sciences, Aristotle University of Thessaloniki, Thessaloniki, Greece; 4 Department of Hygiene, Social-Preventive Medicine and Medical Statistics, School of Medicine, Aristotle University of Thessaloniki, Thessaloniki, Greece; 5 Unit of Reproductive Endocrinology, 1st Department of Obstetrics and Gynecology, Medical School, Aristotle University of Thessaloniki, Thessaloniki, Greece; 6 2nd Department of Neurology, AHEPA Hospital, Faculty of Health Sciences, Aristotle University of Thessaloniki, Thessaloniki, Greece

**Keywords:** systematic review, meta-analysis, chronic periodontitis, severe chronic periodontitis, hemorrhagic stroke

## Abstract

Periodontitis is a chronic, multifactorial inflammatory condition linked to dysbiotic plaque biofilms and characterized by the gradual destruction of the structures supporting the teeth owing to compromised immune system function. Hemorrhagic stroke, which primarily occurs within the brain tissue or in the subarachnoid space as a blood leak of ruptured vessels, is a sudden neurological impairment caused by vascular damage in the central nervous system, resulting in focal neurological deficits. Chronic periodontitis (CP) and hemorrhagic stroke may share common pathogenic features involving inflammation and immune system activation, prompting researchers to investigate their potential connection. The aim of the study is to systematically review the literature on the epidemiological association between CP and hemorrhagic stroke in adults. The study protocol adhered to the PRISMA 2020 guidelines, and the design followed the Cochrane methodology. A thorough literature search encompassing PubMed, Scopus, and Web of Science databases and a manual search and evaluation of gray literature was conducted. Meta-analysis was performed using Review Manager (RevMan) 5.4, with the effect size represented by the odds ratio (OR) and a 95% confidence interval (CI). Heterogeneity was assessed using the chi-squared and *I* ^2^ statistics. The selected articles, written in English without time constraints, focused on observational studies involving patients and controls and included disease diagnostic criteria. Duplicate entries were eliminated. The reliability of each study's results was evaluated using the Newcastle-Ottawa Scale and GRADE tools. Two reviewers conducted the assessments, and a third reviewer resolved any disagreements. The meta-analysis comprised four observational studies involving 1,882 individuals. It revealed that individuals diagnosed with hemorrhagic stroke were notably more likely to have concurrent CP (OR: 6.32; 95% CI: 1.35–29.49; *p* = 0.02) or severe CP (OR: 3.08; 95% CI: 1.56–6.06; *p* = 0.001) compared with healthy controls. A notable occurrence of CP was detected in patients with hemorrhagic stroke compared with controls. Health care professionals need to acknowledge the connection between the two conditions, as it allows them to provide optimal holistic care through a thorough approach to diagnosis and treatment.

## Introduction

Hemorrhagic stroke accounts for approximately 13.5% of all strokes. 1 Hemorrhagic stroke manifests as bleeding in the brain due to a ruptured blood vessel. It can be further subdivided into intracerebral hemorrhage (ICH) and subarachnoid hemorrhage (SAH), which manifest in the brain parenchyma and the subarachnoid space, respectively. 2 ICH is the most common type of hemorrhagic stroke and has been associated with a higher risk of premature mortality and long-term disability. 1 Early diagnosis and treatment are essential, given the usual rapid expansion of hemorrhage, causing sudden deterioration of consciousness and neurological dysfunction. 2 The annual incidence of ICH is 25 cases per 100,000 population, 3 constituting a devastating disease with a high mortality rate of 40 to 50%. 4 At the same time, hemorrhagic stroke is the costliest type of stroke in both high- and middle-income countries. The average cost per patient per year (USD 2020) was estimated at approximately $33,000 in high-income countries, $37,000 in upper-middle-income countries, and $9,000 in low-middle-income countries. 5 An increase in the frequency of ICH is observed in patients with uncontrolled hypertension. Interestingly enough, systemic inflammation response has been associated with the risk of either ischemic or hemorrhagic stroke in elderly patients with hypertension, suggesting its potential as a promising indicator for stroke risk in this population. 6

Chronic periodontitis (CP) is a persistent inflammatory condition of the periodontal tissues caused by anaerobic gram-negative bacteria 7 that involves the gradual destruction of alveolar bone, leading to the development of a periodontal pocket and receding gums. 8 In the beginning, pathogenic bacteria and their byproducts trigger inflammation in the periodontal tissues, causing swelling and bleeding of the gums. 9 At the same time, there is an indirect impact caused by the body's immune response. In particular, the destruction of periodontal tissues occurs due to the activation of monocytes, lymphocytes, fibroblasts, and other defensive cells. 10 Periodontal disease can disturb the natural balance between the oral biofilm microflora and the body's defense mechanisms, leading to its onset. 11 A new classification introduced in 2018 categorizes periodontitis based on its severity (stage) and rate of progression (grade). 12 Advanced periodontitis ranks as the sixth most prevalent disease globally, affecting 10.8% to 11.2% of the population. It significantly contributes to tooth loss, nutritional deficiencies, speech difficulties, diminished self-esteem, and reduced overall quality of life. Furthermore, it is estimated that the global annual economic burden resulting from decreased productivity due to advanced periodontitis amounts to $54 billion. 13 14 CP has been linked to conditions such as cardiovascular disease, specific cancer types, type 2 diabetes, complications during pregnancy, 15 ischemic stroke, 16 and hypertension. 17

Given that periodontal lesions serve as a reservoir for periopathogenic bacteria, CP could potentially be a source of systemic inflammation. 18 This inflammation might play a role in the pathophysiology of cerebral amyloid angiopathy–related hemorrhage. 19 20 Moreover, CP may further potentiate the hemorrhagic stroke–induced systemic inflammatory response syndrome, 21 thus affecting the outcome of the patients' neurological condition. This study is the first attempt to perform a systematic review with meta-analysis exploring the prevalence of CP among patients with hemorrhagic stroke.

## Methods

### Protocol and Registration

The study protocol adhered to the Preferred Reporting Items for Systematic reviews and Meta-Analyses (PRISMA) 2020 guidelines 22 and was registered in the PROSPERO database (Record ID: CRD 42022361022).

### Information Sources

Research studies were sought from three databases (MEDLINE/PubMed, Scopus, and Web of Science) from their inception until September 21, 2024. Additionally, a manual search was conducted on Google and Google Scholar. Gray literature was evaluated through opengrey.eu using the keywords “chronic periodontitis” and “hemorrhagic stroke.” The search strategy employed is detailed in Supplementary Table S1 (available in the online version only).

### Inclusion and Exclusion Criteria

The included studies had to meet the following criteria: (1) they were observational studies (cross-sectional, case-control, or cohort); (2) they were composed in the English language without imposing any limitations related to the time of publication; (3) they had received approval from the ethics committees; (4) they provided precise diagnostic criteria for both CP and hemorrhagic stroke; and (5) they reported data on two distinct study groups: (i) individuals with hemorrhagic stroke and (ii) a control group of healthy individuals.

The diagnosis of CP could be confirmed through clinical and/or imaging observations. Likewise, the diagnosis of hemorrhagic stroke had to be substantiated by clinical and radiographic criteria. Excluded from the analysis were (1) case reports and case series, as they are deemed to provide lower-quality evidence; (2) studies that involved participants below the age of 18 years; and (3) studies that focused on patients with specific conditions, such as malignancies, pregnancy, recent periodontal treatment (scaling, root planning) within the last 6 months, and individuals with fewer than five remaining teeth.

### Study Records

References sourced from electronic databases were systematically cataloged within the Mendeley platform to maintain records of the studies. Duplicate entries were meticulously eliminated, and the retained studies were subsequently migrated to the Rayyan platform, following the methodology outlined by Ouzzani et al. 23 Two reviewers (A.G. and I.T.) independently scrutinized each study's eligibility based on their titles and abstracts. Subsequently, the full texts of the selected studies were also independently assessed by the same two reviewers (A.G. and I.T.). In the event of disagreements arising at any stage of this process, a third reviewer (A.T.) intervened to reach a consensus.

### Data Extraction

Data extraction was performed utilizing Microsoft Excel. A dedicated worksheet was constructed to capture identifying information (comprising the first author's name, publication year, and country) and the demographic attributes of the study populations (including sample size, age distribution, sex distribution, and the number of male and female participants) for each study individually. Furthermore, the criteria for aligning patients and controls, such as gender and age, were meticulously documented.

Concerning hemorrhagic stroke, information about the number of cases and controls was noted, along with the presence or absence of CP and/or severe CP in patients diagnosed with neurological disease. The diagnostic methods and criteria used for disease identification were also documented. As for CP, the number of positive and negative cases within each study's total sample and the diagnostic methods employed were recorded. Two independent reviewers (A.G., I.T.) performed the data extraction, and any discrepancies were resolved by a third reviewer (A.T.).

### Outcomes

The systematic review and meta-analysis focused on documenting chronic and/or severe CP prevalence in hemorrhagic stroke patients and neurologically healthy controls.

### Bias Assessment and Confidence

The quality of observational studies was evaluated using the Newcastle-Ottawa Scale (NOS), as described by Luchini et al. 24 Each study was rated based on the stars earned in the selection, comparability between patients and controls, and exposure (for patient-control studies) and outcome (for cohort and cross-sectional studies). The risk of bias was categorized as “low,” “high,” or “moderate-unclear” independently by two reviewers (A.G., I.T.), with disagreements resolved by a third reviewer (A.T.).

Additionally, the GRADE (Grading of Recommendations Assessment, Development, and Evaluations) tool was utilized to assess the strength of evidence from the studies included in the meta-analysis, following the guidelines outlined by Guyatt et al. 25 Two reviewers (A.G., I.T.) independently evaluated the quality of these studies, classifying them as “high,” “moderate,” “low,” or “very low.” Any conflicts were resolved by a third reviewer (A.T.).

### Statistical Analysis

The meta-analysis of the included studies was conducted using Review Manager (RevMan) 5.4 software. The effect of the outcome, which focused on the presence of CP (a dichotomous variable), was quantified using the odds ratio (OR) with a 95% confidence interval (CI). A random-effects model (inverse variance) was employed for the quantitative synthesis. Heterogeneity was evaluated using the chi-squared and *I* ^2^ statistics.

## Results

**Fig. 1:**
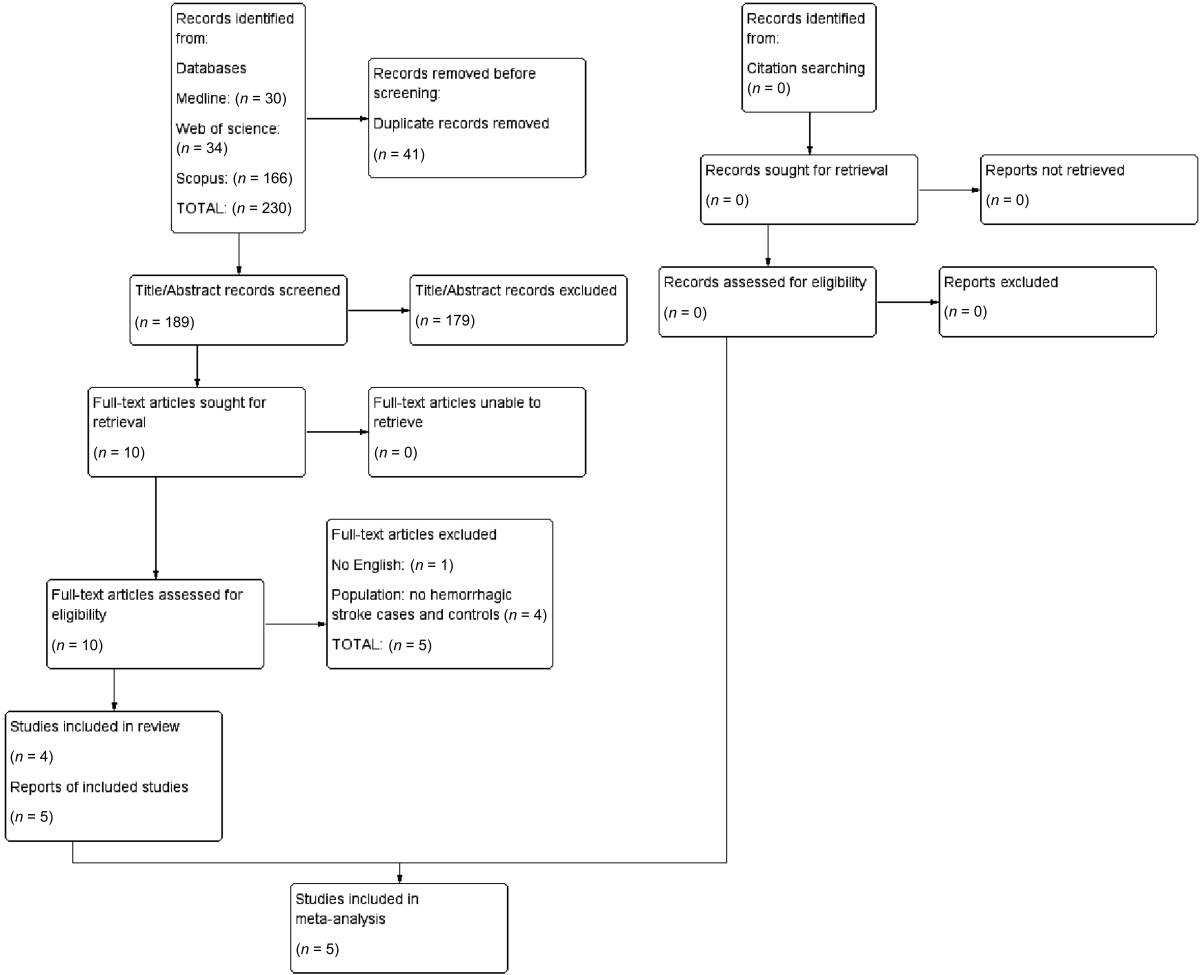
Preferred Reporting Items for Systematic Reviews and Meta-Analyses (PRISMA) 2020 flow diagram of the systematic review and meta-analysis illustrating the study selection process.

The initial literature search yielded 230 studies. After removing duplicates, 189 studies were screened based on their titles and abstracts. Among them, 10 studies were scrutinized as full-text articles. Out of these, five studies were excluded because they did not provide specific numbers of hemorrhagic stroke cases and controls ( *n* = 4) or were written in a language other than English ( *n* = 1). The excluded full-text studies and the reasons for their exclusion are detailed in Supplementary Table S2 (available in the online version only). Ultimately, five studies 26 27 28 29 30 were included in the qualitative and quantitative synthesis (meta-analysis), illustrated in the PRISMA 2020 flow chart ( Fig. 1 ). After contacting the authors, we confirmed that two studies 28 29 analyzed data from the same population sample, so we integrated these studies into the most recent one. 29 The data from the studies included in the meta-analysis are presented in Table 1 .

**Table 1:** Characteristics of the included studies

Study						Population		Chronic periodontitis			Hemorrhagic stroke					Confounding factors taken into account
First author	Year	Country	Type	Matching	*N*	Sex (M/F)	Age (y)	Definition	Positive	Negative	Definition–criteria	Positive	Negative	Perio positive	Perio negative	
Ghizoni 27	2012	Brazil	Case control	Age, sex	67 (7 cases and 60 controls)	N/A	Cases: 59 ± 13 More specific: 54.42 Controls: 48 ± 10	Examining PPD, CAL, BOP, and a dichotomous PLI. Periodontal disease was specifically defined by the presence of at least one site showing a PPD ≥ 4 mm	24	43	Randomly selected patients from the neurosurgery division of the intensive care unit	7	60	7	0	N/A
Kim 26	2010	Korea	Case control	Age, sex	332 (118 cases and 214 controls)	M: 164; F: 168	Cases: 55.19 ± 9.21 Controls: 60.06 ± 11.70	CAL and PPD were selected as a marker	N/A (no and mild periodontitis in the same group)	N/A (no and mild periodontitis in the same group)	Diagnosed hemorrhagic stroke based on the presence of hemorrhagic brain lesions by computed tomography and comprehensive systemic examinations	118	214	*N/A (no and mild periodontitis in the same group), 56 with severe periodontitis	*N/A (no and mild periodontitis in the same group), 276 with moderate, mild or, no periodontitis	Sociodemographic variables, smoking, hypertension, DM, cardiac disease, and BMI
Hallikainen 30	2023	Finland	Case control	Geographically matched	370 (30 cases and 340 controls)	M: 150; F: 220	Cases: 49.0 (31.0–66.0) Controls: 48.0 (30.0–89.0)	Periodontitis was diagnosed taking into account the deepest PPD, categorized as follows: <4 mm no, periodontitis; 4–5 mm, periodontitis; and ≥6 mm, severe periodontitis	207	163	Hospital patients were identified with ICD-0 and procedure codes relevant to aSAH	30	340	23	7	Age, gender, current smoking, caries, periodontitis, and missing teeth
Hallikainen 28	2020	Finland	Case control	Age, sex	103 (33 cases and 70 controls)	M: 35; F: 68	Cases: 50.0 (27.0–76.0) Controls: 57.0 (31.0–76.0)	Patients were classified into three categories according to PPD: <4 mm, no periodontitis; 4–5 mm, periodontitis; and ≥6 mm severe periodontitis. The deepest periodontal PPD in each tooth was taken into account	78	25	Patients referred to the Department of Neurosurgery of Kuopio University Hospital (KUH) for IA treatment	33	70	31	2	Gender, smoking, hypertension, and alcohol abuse
Hallikainen 29	2021	Finland	Case control	N/A	1,010 (24 cases and 986 controls)	Cases: N/A Controls: M: 440; F: 546	N/A	Patients with 4–6 mm PPD together with BOP were diagnosed with periodontitis and those with >6 mm PPD were diagnosed with severe periodontitis	826	184	Patients referred to KUH for the treatment of an uIA ( *n* = 130) or after aSAH ( *n* = 97) were recruited for the study	24	986	24	0	Fender, smoking, and hypertension

Abbreviations: aSAH, aneurysmal subarachnoid hemorrhage; BOP, bleeding on/during probing; CAL, clinical attachment level; DM, diabetes mellitus; F, female; IA, intracranial aneurysm; ICD-10, international Classification of Diseases, 10th Revision; M, male; N/A, not applicable; PLI, plaque index; PPD, probing pocket depth; uIA, unruptured intracranial aneurysms.

### Risk of Bias Assessment

**Fig. 2:**
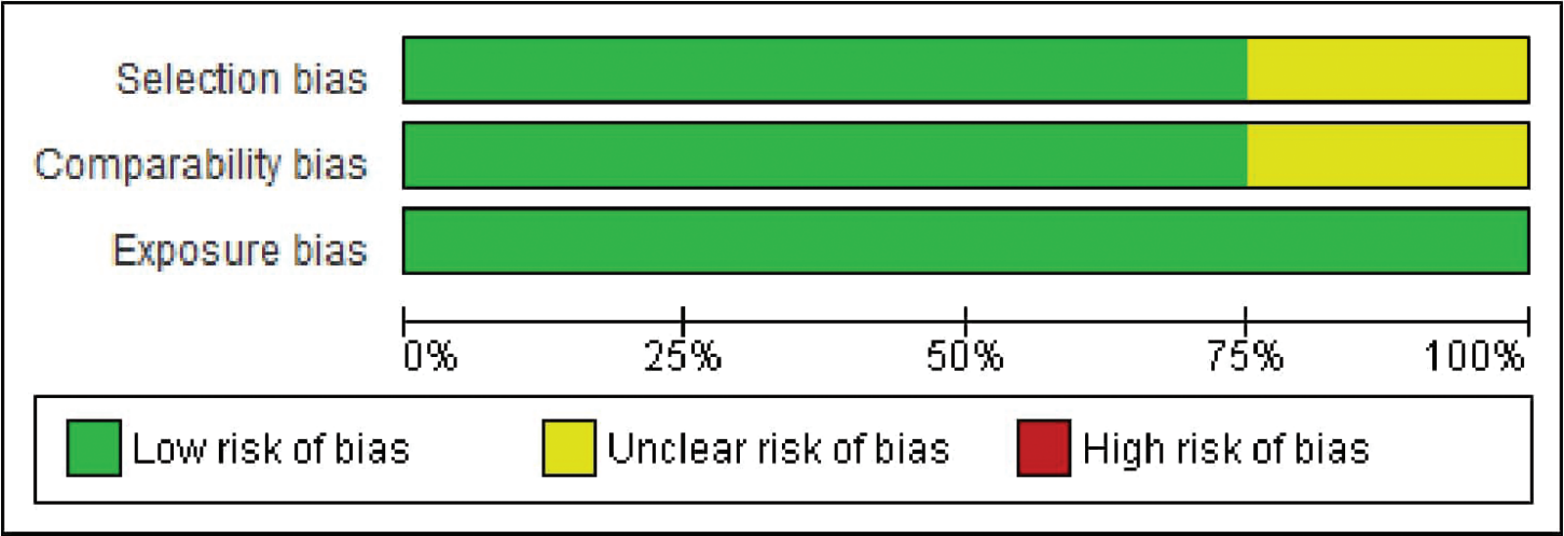
Newcastle-Ottawa Scale (NOS). Risk of bias graph: review authors' judgments about each risk of bias item presented as percentages across all included studies.

**Fig. 3:**
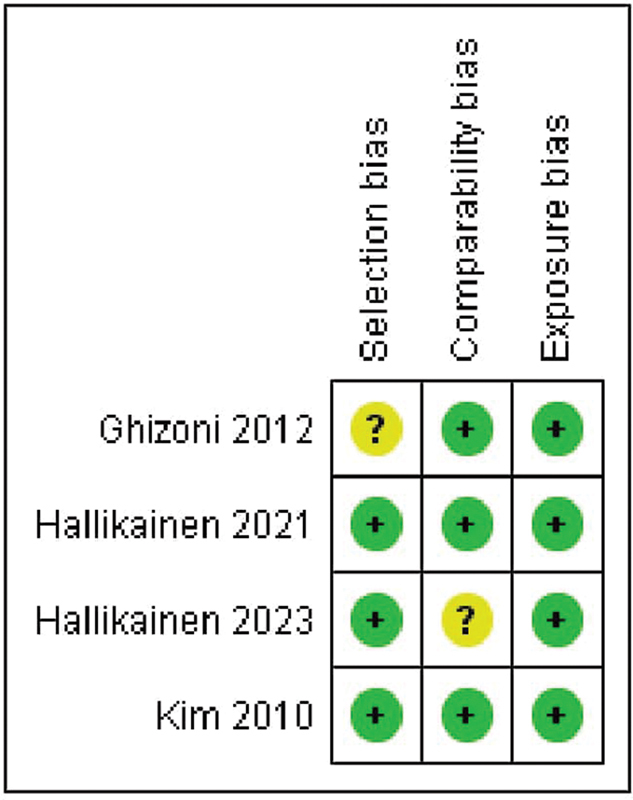
Newcastle-Ottawa Scale (NOS). Risk of bias summary: review authors' judgments about each risk of bias item for each included study.

The NOS was employed to evaluate the quality of the studies included in the analysis. According to this scale, the risk of bias was found to be low, as illustrated in Fig. 2 . A comprehensive graph and evaluation of the bias elements examined using the NOS for each study are depicted in Fig. 3 . Among the studies analyzed in the meta-analysis, three were determined to have a low risk of bias, while one was considered moderate.

### Association between Chronic Periodontitis and Hemorrhagic Stroke

**Fig. 4:**
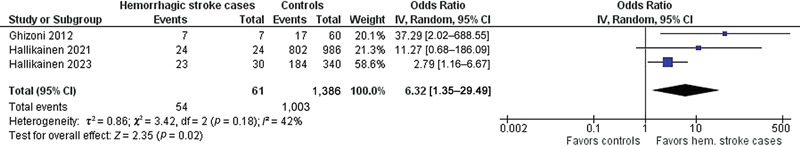
Forest plot demonstrating the association of chronic periodontitis and hemorrhagic stroke. CI, confidence interval; hem., hemorrhagic; IV, inverse variance.

The meta-analysis analyzed data from 1,447 participants, comprising 61 hemorrhagic stroke patients and 1,386 healthy controls. Among them, 54 hemorrhagic stroke patients and 1,003 controls were diagnosed with CP. The analysis revealed significantly higher odds of CP presence in stroke patients than in healthy controls (OR: 6.32; 95% CI: 1.35–29.49; *p* = 0.02; Fig. 4 ). The data exhibited moderate heterogeneity across the studies ( *I* ^2^ = 42%; *p* = 0.18 for heterogeneity; Fig. 4 ).

### Association between Severe Chronic Periodontitis and Hemorrhagic Stroke

**Fig. 5:**
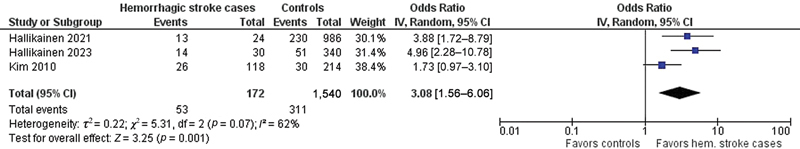
Forest plot demonstrating the association of severe chronic periodontitis and hemorrhagic stroke. CI, confidence interval; hem., hemorrhagic; IV, inverse variance.

The meta-analysis analyzed data from 1,712 participants, comprising 172 hemorrhagic stroke patients and 1,540 healthy controls. Among them, 53 hemorrhagic stroke patients and 311 controls were diagnosed with severe CP. The analysis revealed significantly higher odds of severe CP presence in stroke patients than in healthy controls (OR: 3.08; 95% CI: 1.56–6.06; *p* = 0.001; Fig. 5 ). The data exhibited substantial heterogeneity across the studies ( *I* ^2^ = 62%; *p* = 0.07 for heterogeneity; Fig. 5 ).

### Sensitivity Analyses

Sensitivity analysis was not performed due to the small number of studies. In addition, no study was assessed as having a high risk of bias.

### Evaluation for Publication Bias

The assessment was not conducted due to the limited number of studies. 31

### Strength of Evidence

The GRADE tool was utilized to gauge the robustness of the primary studies incorporated in the meta-analysis. Initially, their rating was low because all the studies included were observational. According to the predefined GRADE criteria for rating the certainty of evidence, one study was classified as high, two studies were classified as moderate, and one as very low ( Table 2 ). Nevertheless, the domains of “inconsistency,” “publication bias,” and “dose–response association” did not apply in this specific context.

**Table 2:** GRADE strength of evidence

	First author	Ghizoni 27	Kim 26	Hallikainen 29	Hallikainen 30
	Year	2012	2010	2021	2023
	Study type	Case control	Case control	Case control	Case control
	Initial rating	Low	Low	Low	Low
	Comparison	Hemorrhagic stroke cases vs. healthy controls	Hemorrhagic stroke cases vs. healthy controls	Hemorrhagic stroke cases vs. healthy controls	Hemorrhagic stroke cases vs. healthy controls
	Outcome	Prevalence of CP	Prevalence of severe CP	Prevalence of CP	Prevalence of CP
Rating down	Study limitations (risk of bias)	Unclear risk (–1)	Low risk (no reason to downgrade)	Low risk (no reason to downgrade)	Low risk (no reason to downgrade)
	Inconsistency	Not applicable (no reason to downgrade)	Not applicable (no reason to downgrade)	Not applicable (no reason to downgrade)	Not applicable (no reason to downgrade)
	Indirectness of evidence	Direct evidence (no reason to downgrade)	Direct evidence (no reason to downgrade)	Direct evidence (no reason to downgrade)	Direct evidence (no reason to downgrade)
	Imprecision	Wide CIs (–1)	Narrow CIs (no reason to downgrade)	Wide CIs but large sample size (no reason to downgrade)	Narrow CIs (no reason to downgrade)
	Publication bias	Not applicable (no reason to upgrade)	Not applicable (no reason to upgrade)	Not applicable (no reason to upgrade)	Not applicable (no reason to upgrade)
Rating up	Magnitude of effect	Strong association (+1)	Moderate association (no reason to upgrade)	Strong association (+1)	Moderate association (no reason to upgrade)
	Dose–response relationship	Not applicable (no reason to upgrade)	Not applicable (no reason to upgrade)	Not applicable (no reason to upgrade)	Not applicable (no reason to upgrade)
	All plausible biases-confounders	Not adjusted for all plausible confounders (no reason to upgrade)	Plausible confounders adjustment (+1)	Plausible confounders adjustment (+1)	Plausible confounders adjustment (+1)
	Final rating	Very low	Moderate	High	Moderate

Abbreviations: CI, confidence interval; CP, chronic periodontitis; GRADE, Grading of Recommendations Assessment, Development, and Evaluations.

## Discussion

The findings of the present study showed a significant association between CP and the prevalence of hemorrhagic stroke. One of the most likely biological mechanisms of association between these two diseases is chronic low-grade systemic inflammation, a state of circulation in the body of various proinflammatory markers established by periodontal lesions. 32 Through various mechanisms, these inflammatory mediators may directly contribute to the risk of stroke. 33 The toxic products of periopathogenic microorganisms, such as lipopolysaccharides (LPS), cause damage to the endothelial cells of the vessels. 34 It is assumed that the inflamed and ulcerated epithelium of the subgingival pocket is a portal of entry into the systemic circulation of both periopathogenic microorganisms and their toxic derivatives. 35 Therefore, etiologically, the chronic presence of periopathogenic microorganisms can lead to atherogenesis through their direct invasion of the arterial wall. 36 37 Periopathogenic organisms such as *Porphyromonas gingivalis* , *Aggregatibacter actinomycetemcomitans* , *Campylobacter rectus* , *Tannerella forsythia* , *Prevotella intermedia* , and *Treponema denticola* have been detected in atheromatous plaques of patients with cardiovascular disease and periodontitis. 38 39 40 Other studies have detected the DNA of oral bacteria in the walls of ruptured and unruptured brain aneurysms. 41 42

Such microorganisms interact with neutrophils, monocytes, and T-lymphocytes, thus producing an acute and chronic inflammatory response. 38 The atherosclerotic process is the blood vessels' immune or metabolic response to harmful agents. 43 Therefore, as inflammatory factors increase and humoral and cellular immunity processes intensify, immune active substances act as harmful elements for the endothelium of blood vessels, facilitating atherosclerotic processes. 44 Consequently, it has been shown that periodontal disease can adversely affect the atherosclerotic process, as it causes an increase in inflammatory mediators such as interleukin-1β (IL-1β), interleukin-6 (IL-6), metalloproteinases (MMP), tumor necrosis factor-α (TNF-α), and C-reactive protein (CRP), due to vascular infiltration by periopathogenic bacteria or due to the establishment of a chronic inflammatory state. 32 45 46 These adverse inflammatory conditions weaken endothelial function, promoting the formation of atheromatous plaques and favoring their rupture. The structural integrity of atherosclerotic plaques is thus compromised through the induction of vascular instability, which leads to increased susceptibility to ischemic and hemorrhagic manifestations. 47 Tonetti et al 48 demonstrated that while intensive periodontal treatment directly led to acute, short-term systemic inflammation and endothelial dysfunction, 6 months after treatment, oral health benefits were associated with improved endothelial function. Along with these, increased oxidative stress 49 and proteolytic activities in blood vessels due to periodontal disease can affect the processes of their rupture. 50 Given that periodontitis is an activation factor of the inflammatory mechanism, as it accelerates the activation and recruitment of circulating neutrophils and monocytes, it seems to contribute to the structural change of the wall of cerebral arteries, predisposing them to the development and rupture of aneurysms. 28

Moreover, some important risk factors are common to both hemorrhagic stroke and periodontitis. In particular, hypertension 51 and diabetes mellitus, 52 53 as diseases that have been documented to be associated with periodontitis, may be potential cofactors of its association with stroke. Evidence from a randomized controlled trial by D'Aiuto et al 54 documented that intensive periodontal treatment improves the lipid profile while reducing systemic inflammatory markers and high blood pressure. It is reasonable to expect that effective treatment of periodontal disease could contribute to reducing the incidence of hemorrhagic stroke.

Due to tooth loss, patients with periodontal disease often wear removable prosthetic restorations, which can cause masticatory dysfunction, as the use of removable dental prosthetics is often accompanied by a reduced ability to chew as a consequence of mucosal elasticity. 55 These patients choose soft foods, which often contain more carbohydrates and fat, resulting in their tendency toward obesity, while full denture wearers may overuse salt and sugar due to a reduced sense of taste. 56 These bad eating habits might, therefore, be directly or indirectly aggravating factors for the occurrence of hemorrhagic stroke.

This study's robustness stems from its thorough exploration of the literature, encompassing both published and gray materials. Additionally, it adheres to the latest PRISMA 2020 research protocol and employs dependable tools to evaluate the primary studies' data integrity. Also, to our knowledge, this study is the first systematic review and meta-analysis to investigate the epidemiological association between CP and hemorrhagic stroke specifically rather than “stroke” in general. Furthermore, the association of severe CP with hemorrhagic stroke is evaluated separately for the first time. Another notable aspect of this review is its exclusive emphasis on CP, distinct from a broad evaluation of periodontal diseases like gingivitis. This specific focus allows for an in-depth analysis of the long-term impact that established periodontal inflammation might have on neurological diseases.

This study also acknowledges certain limitations. The included primary studies are observational (no randomized control studies were available), lacking robust evidence and failing to establish any direct cause-and-effect relationship between the two diseases. Moreover, the studies included in the analysis vary in sample sizes, which introduces methodological challenges when interpreting the meta-analysis results. The study by Ghizoni et al 27 was based on data from very few cases and might be the reason for such a high effect magnitude. Also, in the same study, no confounders have been adjusted. Furthermore, certain confounding factors were not thoroughly examined in the primary studies analyzed. For instance, the impact of socioeconomic status is expected to be significant in the relationship between CP and hemorrhagic stroke. An additional confounding factor not examined in the primary studies is the individual health beliefs of each patient. Individuals who do not prioritize their health may be more likely to experience uncontrolled metabolic conditions such as hypertension, diabetes, and obesity, which can increase the risk of stroke. Furthermore, these health issues may contribute to poor oral hygiene, increasing the likelihood of developing CP.

Moreover, the limited number of chosen studies restricts the potential for further analyses, such as evaluating publication bias using a funnel plot. Investigating the subtype of hemorrhagic stroke proved challenging due to the distinct pathophysiology of ICH and SAH. Subgroup analysis was not feasible as the primary studies used the broad term “hemorrhagic stroke” to describe the condition. Additionally, some GRADE domains were not applicable for assessment, diminishing the tool's reliability and usefulness in this context.

Due to the limited available data, conducting a high-quality meta-analysis of observational studies could offer substantial evidence and highlight specific research areas that demand further exploration. Such an area could include the paradoxical result of the present study that CP, in general, is more likely to be associated with hemorrhagic stroke (OR: 6.32) than severe CP (OR: 3.08).

In summary, there is a statistically significant increase in the prevalence of CP among individuals diagnosed with hemorrhagic stroke when compared with their healthy counterparts. Health care professionals must acknowledge this association to provide top-quality care, employing comprehensive diagnostic and therapeutic strategies.

## References

[1] Sacco S Marini C Toni D Olivieri L Carolei A Incidence and 10-year survival of intracerebral hemorrhage in a population-based registry Stroke 2009 40 02 394 399 19038914 10.1161/STROKEAHA.108.523209

[2] American Heart Association Council on Epidemiology and Prevention Statistics Committee and Stroke Statistics Subcommittee Virani S S Alonso A Benjamin E J Heart Disease and Stroke Statistics-2020 update: a report from the American Heart Association Circulation 2020 141 09 e139 e596 31992061 10.1161/CIR.0000000000000757

[3] Koivunen R J Satopää J Meretoja A Incidence, risk factors, etiology, severity and short-term outcome of non-traumatic intracerebral hemorrhage in young adults Eur J Neurol 2015 22 01 123 132 25142530 10.1111/ene.12543

[4] Yan F Yi Z Hua Y Predictors of mortality and recurrent stroke within five years of intracerebral hemorrhage Neurol Res 2018 40 06 466 472 30134784 10.1080/01616412.2018.1451266

[5] Strilciuc S Grad D A Radu C The economic burden of stroke: a systematic review of cost of illness studies J Med Life 2021 14 05 606 619 35027963 10.25122/jml-2021-0361 PMC8742896

[6] Cai X Song S Hu J Systemic inflammation response index as a predictor of stroke risk in elderly patients with hypertension: a cohort study J Inflamm Res 2023 16 4821 4832 37901383 10.2147/JIR.S433190 PMC10612501

[7] Nair S Faizuddin M Dharmapalan J Role of autoimmune responses in periodontal disease Autoimmune Dis 2014 2014 596824 24963400 10.1155/2014/596824 PMC4055614

[8] Cekici A Kantarci A Hasturk H Van Dyke T E Inflammatory and immune pathways in the pathogenesis of periodontal disease Periodontol 2000 2014 64 01 57 80 24320956 10.1111/prd.12002 PMC4500791

[9] Loos B G Van Dyke T E The role of inflammation and genetics in periodontal disease Periodontol 2000 2020 83 01 26 39 32385877 10.1111/prd.12297 PMC7319430

[10] Wielento A Lagosz-Cwik K B Potempa J Grabiec A M The role of gingival fibroblasts in the pathogenesis of periodontitis J Dent Res 2023 102 05 489 496 36883660 10.1177/00220345231151921 PMC10249005

[11] Socransky S S Haffajee A D Dental biofilms: difficult therapeutic targets Periodontol 2000 2002 28 12 55 12013340 10.1034/j.1600-0757.2002.280102.x

[12] Papapanou P N Sanz M Buduneli N Periodontitis: consensus report of workgroup 2 of the 2017 World Workshop on the Classification of Periodontal and Peri-Implant Diseases and Conditions J Periodontol 2018 89 01 S173 S182 29926951 10.1002/JPER.17-0721

[13] Frencken J E Sharma P Stenhouse L Green D Laverty D Dietrich T Global epidemiology of dental caries and severe periodontitis: a comprehensive review J Clin Periodontol 2017 44 18 S94 S105 28266116 10.1111/jcpe.12677

[14] Tonetti M S Jepsen S Jin L Otomo-Corgel J Impact of the global burden of periodontal diseases on health, nutrition and wellbeing of mankind: a call for global action J Clin Periodontol 2017 44 05 456 462 28419559 10.1111/jcpe.12732

[15] Bui F Q Almeida-da-Silva C LC Huynh B Association between periodontal pathogens and systemic disease Biomed J 2019 42 01 27 35 30987702 10.1016/j.bj.2018.12.001 PMC6468093

[16] Fagundes N CF Almeida A PCPSC Vilhena K FB Magno M B Maia L C Lima R R Periodontitis as a risk factor for stroke: a systematic review and meta-analysis Vasc Health Risk Manag 2019 15 519 532 31806984 10.2147/VHRM.S204097 PMC6847992

[17] Italian working group on Hypertension, Periodontitis (Hy-Per Group) Del Pinto R Landi L Grassi G Hypertension and periodontitis: a joint report by the Italian Society of Hypertension (SIIA) and the Italian Society of Periodontology and Implantology (SIdP) High Blood Press Cardiovasc Prev 2021 28 05 427 438 34562228 10.1007/s40292-021-00466-6 PMC8484186

[18] Cho M J Kim Y S Park E Y Kim E K Association between periodontal health and stroke: results from the 2013-2015 Korea National Health and Nutrition Examination Survey (KNHANES) J Dent Sci 2021 16 01 268 274 33384808 10.1016/j.jds.2020.05.006 PMC7770241

[19] Ohashi S N DeLong J H Kozberg M G Role of inflammatory processes in hemorrhagic stroke Stroke 2023 54 02 605 619 36601948 10.1161/STROKEAHA.122.037155

[20] de Souza A Tasker K Inflammatory cerebral amyloid angiopathy: a broad clinical spectrum J Clin Neurol 2023 19 03 230 241 37151140 10.3988/jcn.2022.0493 PMC10169922

[21] Li X Chen G CNS-peripheral immune interactions in hemorrhagic stroke J Cereb Blood Flow Metab 2023 43 02 185 197 36476130 10.1177/0271678X221145089 PMC9903219

[22] Page M J McKenzie J E Bossuyt P M The PRISMA 2020 statement: an updated guideline for reporting systematic reviews BMJ 2021 372 07 n71 33782057 10.1136/bmj.n71 PMC8005924

[23] Ouzzani M Hammady H Fedorowicz Z Elmagarmid A Rayyan-a web and mobile app for systematic reviews Syst Rev 2016 5 01 210 27919275 10.1186/s13643-016-0384-4 PMC5139140

[24] Luchini C Stubbs B Solmi M Assessing the quality of studies in meta-analyses: advantages and limitations of the Newcastle Ottawa Scale World J Metaanal 2017 5 80 84

[25] Guyatt G Oxman A D Akl E A GRADE guidelines: 1. Introduction-GRADE evidence profiles and summary of findings tables J Clin Epidemiol 2011 64 04 383 394 21195583 10.1016/j.jclinepi.2010.04.026

[26] Kim H D Sim S J Moon J Y Hong Y C Han D H Association between periodontitis and hemorrhagic stroke among Koreans: a case-control study J Periodontol 2010 81 05 658 665 20429645 10.1902/jop.2010.090614

[27] Ghizoni J S Taveira L A Garlet G P Increased levels of *Porphyromonas gingivalis* are associated with ischemic and hemorrhagic cerebrovascular disease in humans: an *in vivo* study J Appl Oral Sci 2012 20 01 104 112 22437687 10.1590/S1678-77572012000100019 PMC3928781

[28] Hallikainen J Lindgren A Savolainen J Periodontitis and gingival bleeding associate with intracranial aneurysms and risk of aneurysmal subarachnoid hemorrhage Neurosurg Rev 2020 43 02 669 679 30972514 10.1007/s10143-019-01097-1 PMC7186244

[29] Hallikainen J Pyysalo M Keränen S Systemic immune response against the oral pathogens *Porphyromonas gingivalis* and *Aggregatibacter actinomycetemcomitans* is associated with the formation and rupture of intracranial aneurysms Eur J Neurol 2021 28 09 3089 3099 34145948 10.1111/ene.14986

[30] Hallikainen J Pessi T Vehkalahti M Suominen A L Pyysalo M Frösen J Unlike severe periodontitis, caries does not associate with intracranial aneurysms or aneurysmal subarachnoid hemorrhage Acta Neurochir (Wien) 2023 165 01 169 175 36416942 10.1007/s00701-022-05406-4 PMC9840572

[31] Sterne J A Sutton A J Ioannidis J P Recommendations for examining and interpreting funnel plot asymmetry in meta-analyses of randomised controlled trials BMJ 2011 343 d4002 21784880 10.1136/bmj.d4002

[32] Cecoro G Annunziata M Iuorio M T Nastri L Guida L Periodontitis, low-grade inflammation and systemic health: a scoping review Medicina (Kaunas) 2020 56 06 272 32486269 10.3390/medicina56060272 PMC7353850

[33] Libby P Inflammation in atherosclerosis Arterioscler Thromb Vasc Biol 2012 32 09 2045 2051 22895665 10.1161/ATVBAHA.108.179705 PMC3422754

[34] Gurav A N The implication of periodontitis in vascular endothelial dysfunction Eur J Clin Invest 2014 44 10 1000 1009 25104241 10.1111/eci.12322

[35] Leira Y Rodríguez-Yáñez M Arias S Periodontitis as a risk indicator and predictor of poor outcome for lacunar infarct J Clin Periodontol 2019 46 01 20 30 30362631 10.1111/jcpe.13032

[36] Haraszthy V I Zambon J J Trevisan M Zeid M Genco R J Identification of periodontal pathogens in atheromatous plaques J Periodontol 2000 71 10 1554 1560 11063387 10.1902/jop.2000.71.10.1554

[37] Fiehn N E Larsen T Christiansen N Holmstrup P Schroeder T V Identification of periodontal pathogens in atherosclerotic vessels J Periodontol 2005 76 05 731 736 15898933 10.1902/jop.2005.76.5.731

[38] Deshpande R G Khan M B Genco C A Invasion of aortic and heart endothelial cells by *Porphyromonas gingivalis* Infect Immun 1998 66 11 5337 5343 9784541 10.1128/iai.66.11.5337-5343.1998 PMC108667

[39] Atarbashi-Moghadam F Havaei S R Havaei S A Hosseini N S Behdadmehr G Atarbashi-Moghadam S Periopathogens in atherosclerotic plaques of patients with both cardiovascular disease and chronic periodontitis ARYA Atheroscler 2018 14 02 53 57 30108636 10.22122/arya.v14i2.1504 PMC6087625

[40] Pavlic V Peric D Kalezic I S Identification of periopathogens in atheromatous plaques obtained from carotid and coronary arteries BioMed Res Int 2021 2021 9.986375E6 10.1155/2021/9986375 PMC8225426 34222492

[41] Pyysalo M J Pyysalo L M Pessi T Karhunen P J Öhman J E The connection between ruptured cerebral aneurysms and odontogenic bacteria J Neurol Neurosurg Psychiatry 2013 84 11 1214 1218 23761916 10.1136/jnnp-2012-304635

[42] Pyysalo M J Pyysalo L M Pessi T Bacterial DNA findings in ruptured and unruptured intracranial aneurysms Acta Odontol Scand 2016 74 04 315 320 26777430 10.3109/00016357.2015.1130854

[43] Rafieian-Kopaei M Setorki M Doudi M Baradaran A Nasri H Atherosclerosis: process, indicators, risk factors and new hopes Int J Prev Med 2014 5 08 927 946 25489440 PMC4258672

[44] Harangi M Szodoray P Paragh G Atherosclerosis: a complex interplay of inflammatory processes Clin Lipidol 2009 4 167 187

[45] Henderson B Nair S P Ward J M Wilson M Molecular pathogenicity of the oral opportunistic pathogen *Actinobacillus actinomycetemcomitans* Annu Rev Microbiol 2003 57 29 55 14527274 10.1146/annurev.micro.57.030502.090908

[46] Paraskevas S Huizinga J D Loos B G A systematic review and meta-analyses on C-reactive protein in relation to periodontitis J Clin Periodontol 2008 35 04 277 290 18294231 10.1111/j.1600-051X.2007.01173.x

[47] Hansson G K Robertson A K Söderberg-Nauclér C Inflammation and atherosclerosis Annu Rev Pathol 2006 1 297 329 18039117 10.1146/annurev.pathol.1.110304.100100

[48] Tonetti M S D'Aiuto F Nibali L Treatment of periodontitis and endothelial function N Engl J Med 2007 356 09 911 920 17329698 10.1056/NEJMoa063186

[49] Dursun E Akalin F A Genc T Cinar N Erel O Yildiz B O Oxidative stress and periodontal disease in obesity Medicine (Baltimore) 2016 95 12 e3136 27015191 10.1097/MD.0000000000003136 PMC4998386

[50] Söder P O Meurman J H Jogestrand T Nowak J Söder B Matrix metalloproteinase-9 and tissue inhibitor of matrix metalloproteinase-1 in blood as markers for early atherosclerosis in subjects with chronic periodontitis J Periodontal Res 2009 44 04 452 458 18973519 10.1111/j.1600-0765.2008.01145.x

[51] Martin-Cabezas R Seelam N Petit C Association between periodontitis and arterial hypertension: a systematic review and meta-analysis Am Heart J 2016 180 98 112 27659888 10.1016/j.ahj.2016.07.018

[52] Preshaw P M Alba A L Herrera D Periodontitis and diabetes: a two-way relationship Diabetologia 2012 55 01 21 31 22057194 10.1007/s00125-011-2342-y PMC3228943

[53] Casanova L Hughes F J Preshaw P M Diabetes and periodontal disease: a two-way relationship Br Dent J 2014 217 08 433 437 25342350 10.1038/sj.bdj.2014.907

[54] D'Aiuto F Parkar M Nibali L Suvan J Lessem J Tonetti M S Periodontal infections cause changes in traditional and novel cardiovascular risk factors: results from a randomized controlled clinical trial Am Heart J 2006 151 05 977 984 16644317 10.1016/j.ahj.2005.06.018

[55] Liedberg B Stoltze K Owall B The masticatory handicap of wearing removable dentures in elderly men Gerodontology 2005 22 01 10 16 15747893 10.1111/j.1741-2358.2004.00050.x

[56] Károlyházy K Arányi Z Hermann P Vastagh I Márton K Oral health status of stroke patients related to residual symptoms: a case-control epidemiological study in Hungary Oral Health Prev Dent 2018 16 03 233 239 29946578 10.3290/j.ohpd.a40672

