# Supplementary material for: Association of Chronic Periodontitis with Hemorrhagic Stroke: A Systematic Review and Meta-Analysis
Source: Eur J Dent. 2024 Dec 10;19(2):265–74. doi: 10.1055/s-0044-1793844 (PMC12020598; doi:10.1055/s-0044-1793844)
Supplement: Supplementary file 1 — Supplementary Material [file 10-1055-s-0044-1793844-s2433417.pdf]

Supplementary Table S1 Search strategy in MEDLINE

Query
(((((((((("generalized periodontitis") OR ("chronic periodontal inflammation")) OR (periodontitis)) OR ("chronic periodontitis")) OR ("mild periodontal disease")) OR ("moderate periodontal disease")) OR ("severe periodontal disease")) OR ("periodontal disease")) OR (periodontal disease[MeSH Terms])) OR (periodontitis[MeSH Terms])) OR (chronic periodontitis[MeSH Terms])) AND ((((("hemorrhagic stroke") OR ("brain hemorrhage")) OR ("cerebral hemorrhage")) OR ("intracerebral hemorrhage")) OR ("subarachnoid hemorrhage"))

Results: 30

Search strategy in Scopus

Query
((TITLE-ABS-KEY (generalized AND periodontitis) OR TITLE-ABS-KEY (chronic AND periodontal AND inflammation) OR TITLE-ABS-KEY (periodontitis) OR TITLE-ABS-KEY (chronic AND periodontitis) OR TITLE-ABS-KEY (mild AND periodontal AND disease) OR TITLE-ABS-KEY (moderate AND periodontal AND disease) OR TITLE-ABS-KEY (severe AND periodontal AND disease) OR TITLE-ABS-KEY (periodontal AND disease))) AND ((TITLE-ABS-KEY (hemorrhagic AND stroke) OR TITLE-ABS-KEY (brain AND hemorrhage) OR TITLE-ABS-KEY (cerebral AND hemorrhage) OR TITLE-ABS-KEY (intracerebral AND hemorrhage) OR TITLE-ABS-KEY (subarachnoid AND hemorrhage)))

Results: 166

Search strategy in Web of Science

Query
#1: generalized periodontitis (All Fields) or chronic periodontal inflammation (All Fields) or periodontitis (All Fields) or chronic periodontitis (All Fields) or mild periodontal disease (All Fields) or moderate periodontal disease (All Fields) or severe periodontal disease (All Fields) or periodontal disease (All Fields)<br>#2: ((((ALL = (hemorrhagic stroke)) OR ALL = (brain hemorrhage)) OR ALL = (cerebral hemorrhage)) OR ALL = (intracerebral hemorrhage)) OR ALL = (subarachnoid hemorrhage)<br>#3: #1 AND #2

Results: 34.

Supplementary Table S2 List of excluded studies with rationale

ID	First author, year	Reason for exclusion
1	Shirotani, 2005	No English language
2	Byun, 2020	Population: no hemorrhagic stroke cases and controls
3	Patel, 2020	Population: no hemorrhagic stroke cases and controls
4	Diouf, 2015	Population: no hemorrhagic stroke cases and controls
5	Budin, 2014	Population: no hemorrhagic stroke cases and controls

 © 2024. The Author(s).
